# Gender differences in cardiac rehabilitation participation and outcomes: an 18-year retrospective study in Iran

**DOI:** 10.1186/s43044-024-00565-4

**Published:** 2024-10-04

**Authors:** Marzieh Najafi, Zahra Teimouri-Jervekani, Marjan Jamalian, Hamidreza Roohafza, Mohammad Hossein Paknahad, Mohammad mahdi Hadavi, Neda Dorostkar, Masoumeh Sadeghi

**Affiliations:** 1 Department of Cardiology, Isfahan Cardiovascular Research Center, Cardiovascular Research Institute, Chamran Cardiovascular Medical and Research Hospital, Isfahan University of Medical Sciences, Isfahan, Iran; 2 Cardiac Rehabilitation Research Center, Cardiovascular Research Institute, Isfahan University of Medical Sciences, Isfahan, Iran; 3 Heart Failure Research Center, Cardiovascular Research Institute, Isfahan University of Medical Sciences, Isfahan, Iran; 4 Isfahan Cardiovascular Research Center, Cardiovascular Research Institute, Isfahan University of Medical Sciences, Isfahan, Iran; 5 Interventional Cardiology Research Center, Cardiovascular Research Institute, Isfahan University of Medical Sciences, Isfahan, Iran

**Keywords:** Cardiac Rehabilitation, Percutaneous coronary intervention, Coronary artery bypass grafting, Coronary heart diseases, Psychological factors, Risk factor

## Abstract

**Background:**

Cardiac rehabilitation (CR) is crucial for addressing cardiovascular diseases globally, with a specific emphasis on gender differences. Despite its demonstrated benefits for women, there's limited acceptance globally, especially in low- and middle-income countries. The program aims to optimize risk factors and improve overall patient well-being.

**Methods:**

A cohort study was performed on those who were candidates for CR programs during 2001–2019. Assessments were performed within one week before and one week after the 8-week CR program. Age, sex, smoking status, clinical data, resting systolic and diastolic blood pressure (SBP and DBP, respectively), echocardiography and laboratory data were obtained. Functional capacity was evaluated using the international physical activity questionnaire, and a treadmill exercise test. Anxiety, depression, general quality of life (QoL), and health-related QoL were selected for psychological status. Then statistical analysis was performed on data.

**Result:**

In this study, the number of male patients was 1526 (73.69%). The average age of patients in the female group was higher than that of males (58.66 ± 9.08 vs. 56.18 ± 9.94), according to the crude model results, the changes in emotional, social and physical scores were significant (*P*-value:0.028, 0.018, 0.030), as well as the differences in Mets and smoking were significant (*P*-value for both < 0.001) in the adjusted model, the emotional variables and Mets changes were significant in two groups, so that the emotional score in the female group was higher than that of the male group, and the female Mets score was significantly lower than that of the male group.

**Conclusion:**

The CR program can improve cardiovascular outcomes, but the greatest impact was on the quality of life, patient METs and smoking behavers. Also the number of female participants in the CR program was less than the number of males.

## Background

According to disability-adjusted life years (DALYs), cardiovascular diseases (CVDs) are major causes of death and disability worldwide. With significant sex differences between males and females in the pathophysiology, presentation, and management of the disease [1–3].

Cardiac rehabilitation (CR) as a treatment strategy contains a multidimensional approach involving exercise training, improving physical activity status, providing health education, managing cardiovascular risks as secondary prevention, and offering psychological support. It is responsible for meeting the specific needs of individuals diagnosed with CVD (such as myocardial infarction (MI), stable chronic heart failure, stable angina, cardiac arrhythmias such as atrial fibrillation) or those patients who have cardiac procedures like coronary artery bypass surgery (CABG), percutaneous coronary interventions(PCI), heart valve repair and heart transplantation field [4–6]. Emphasis on enhancing overall patient well-being and improving quality of life in addition to enhancing cardiovascular outcomes and secondary prevention are some goals of contemporary cardiac rehabilitation. Furthermore, optimizing CVD risk factors such as serum lipid profiles, blood glucose levels, body weight, and blood pressure is another positive outcome field [7, 8].

Even though 70–80% of deaths due to cardiovascular diseases are in low- and middle-income countries and the Middle East, unfortunately, novel CR program faces limited acceptance globally and in these countries it is estimated that around 23% of patients receive these services, comparing to USA with a program per 102000 people in low income countries this statistics fall to a program per 1–6 million people [9–11].

Considering the biological, psychological, and social distinctions between males and females, evaluating the effect of CR on risk factors and the level of engagement for both genders is crucial. Additionally, these gender differences persisted even after adjusting for age, as a critical factor being given that females often experience cardiovascular events in older age. Age variations are widespread in CR participation [12]. In addition, despite greater potential clinical needs in women (such as psychosocial challenges like depression, comorbidities and functional capacity they enroll less to these programs. Hence these reasons emphasize the demonstrated benefits of CR for women [1]. Of course, the gender difference in CR participants is different based on the geographical region, for example, in Iran, as a country with a high level of gender inequality, this statistic is much higher than in USA or Europe [13].

This study aims to evaluate the participation of men and women in a Cardiac Rehabilitation program using an 18-year registry from the Isfahan Cardiac Rehabilitation Centre. By understanding gender-specific patterns in Iran, we can identify barriers and develop targeted interventions to promote equal access to CR for both sexes. Additionally, our findings may provide valuable insights for other low- and middle-income countries facing similar challenges, helping to inform global efforts to reduce health disparities and improve CR participation rates.

## Methods

### Patients

This was a cohort study that was performed at the Cardiac Rehabilitation Center of Isfahan Cardiovascular Research Institute, Isfahan, Iran. The study population consisted of cardiac patients referred to the CR unit of Chamran Hospital in Isfahan, Iran, during 2001–2019.

An inclusion criterion was referring to the CR center by a cardiologist and having the ability and satisfaction to participate in the CR program. The exclusion criteria are listed as patients with serious medical conditions (e.g., cerebral vascular attacks, chronic kidney disease, cirrhosis, and chronic obstructive sleep apnea), patients who could not tolerate physical activity sessions, > 20% missing data in the medical documents or questionnaires, a previous history of PCI or CABG, and missing two or more CR program sessions.

It has to be mentioned that these patients with exclusion criteria had been excluded before the gathering data.

Data were gathered considering patients’ medical records. The sample size is all the files of Chamran Cardiac Rehabilitation Centre from 2001 to 2019. Demographic characteristics (such as age, sex, height, weight, marriage, education, occupation, economic, social status, etc.) and extracted data from patients' records (such as underlying heart disease, medical history, level of physical activity, blood pressure and initial tests) were analyzed via descriptive statistics.

### Protocol

IHD patients who have been referred to a CR center for rehabilitation first fill out pre-prepared questionnaires. These questionnaires include the demographic information of patients such as BMI, smoking, and physical activity. According to the questionnaires, Quality of life (Emotional, social, and physical) were evaluated. Psychological parameters were assessed by IPAQ score. Patients whose level of stress, anxiety, or depression in the psychiatric questionnaire (based on IPAQ protocol) is severe or moderate to severe are referred to a psychiatrist for treatment to receive psychotherapy or medication. Then blood samples were taken from all patients and sent to the laboratory to measure the desired parameters (FBS, TG, cholesterol, LDL, HDL). Exercise testing is performed on all patients and patients' METs are determined. Based on the functional capacity of patients, their appropriate exercises were selected, and patients participated in 24 rehabilitation sessions for 2 months. Rehabilitation sessions last about 1 h. At the beginning of the session, patients' vital signs are measured and recorded, and then their vital signs were monitored throughout the session. At the end of rehabilitation sessions, patients were re-examined for exercise and their functional capacity, and the amount of stress and anxiety. Patients were given the necessary training for physical activity at home, and their functional capacity was re-evaluated after rehabilitation. Finally, all data collected from patients were analyzed.

### Statistical analysis

In this study, Quantitative data were evaluated using means and standard deviations. Numbers and percentages were used to display numerical data. Quantitative data were compared using the independent t test. Where appropriate, categorical data were compared using Fisher’s exact test or Chi-square analysis. For comparison pre and post in groups used paired-t test. Adjusted and non-adjusted Linear and logistic regression analysis was performed for pre–post changes of parameters and computed with 95% confidence intervals. Each statistical test has two sides. For the statistical study, IBM, Armonk, New York, USA, SPSS version 22 was utilized. Significant *P* values were those with a value of less than 0.05.

## Results

The analysis sample included 2064 patients, 1521 (73.69%) males, and 543 (26.31%) females.

Table 1 shows the socio-demographic characteristics, the rates of comorbidities, the type of CVD intervention, and the cardiac history of the participants.

**Table 1:** Patients’ socio-demographic characteristics, comorbidities, type of cardiac intervention, and participation in CR sessions

Variables	Total (*n* = 2064)	Male (*n* = 1521)	Female (*n* = 543)	*P* value
*Demographics factors*				
Age (mean ± SD)	56.83 ± 9.78	56.18 ± 9.94	58.66 ± 9.08	< 0.001
*Marital status*				
Single	352 (17.05%)	281 (18.50%)	71 (13.08%)	< 0.001
Married	1573 (76.21%)	1223 (80.40%)	350 (64.45%)	
Divorce/Death of partner	139 (6.73%)	17 (1.10%)	122 (22.47%)	
*Educational state*				
0–5 years	987 (47.82%)	586 (38.5%)	401 (73.85%)	< 0.001
6–12 years	647 (31.35%)	543 (35.70%)	104 (19.15%)	
> 12 years	430 (20.83%)	392 (25.80%)	38 (7.00%)	
*Occupation*				
Self-employment + labor	566 (27.42%)	552 (36.3%)	14 (2.60%)	< 0.001
Employee	409 (19.82%)	389 (25.6%)	20 (3.7%)	
Housewife	462 (22.38%)	0 (0%)	462 (85%)	
Unemployed (retired and retired employed)	627 (30.38%)	580 (38.1%)	47 (8.7%)	
*Comorbidity*				
Hypertension (HTN)	655 (31.73%)	411 (27.02%)	244 (44.93%)	< 0.001
Diabetes Mellitus (DM)	528 (25.58%)	337 (22.16%)	191 (35.17%)	< 0.001
Hyperlipidemia	900 (43.60%)	599 (39.38%)	301 (55.43%)	< 0.001
*Cardiac intervention*				
PCI	934 (45.26%)	728 (47.9%)	206 (37.8%)	< 0.001
CABG	732 (35.46%)	538 (35.4%)	194 (35.7%)	
Number of session	18.73 ± 7.24	18.95 ± 7.10	18.16 ± 7.58	0.048

### Age, marital state

The mean (± SD) age of the participants was 56.83 ± 9.78 in total, 58.66 ± 9.08 in women, and 56.18 ± 9.94 in men. Males were most likely to be married, and the differences were significant (*P* < 0.001). (Table 1).

### Comorbidities

Prevalence of hypertension (HTN), hyperlipidemia and even DM were significantly higher in females (44.93%, 55.43% & 35.17%) compared to males (27.02%, 39.38% & 22.16%), respectively (*P* < 0.001); (Table 1).

### Cardiac intervention

In terms of cardiac interventions significant differences were seen for PCI. PCI was significantly higher in men (47.9%) than women (37.8%) (*P* < 0.001) (Table 1).

### Participation in CR sessions

Participation in CR sessions was significantly higher in men 18.95 ± 7.10 compared with women 18.16 ± 7.58 (*P* = 0.48 < 0.05). Although the difference was significant it was not remarkable. (Table 1).

Table 2 shows variables in 4 separates groups:

**Table 2:** Includes the investigated variables in three categories of laboratory, psychological and clinical variables in men and women and comparing their changes before and after participating in rehabilitation sessions

	Pre			Post			Difference post–pre		
	Male	Female	*P* value	Male	Female	*p* value	Male	Female	*P* value^+^
*Lifestyle*									
BMI	26.70 ± 3.61	29.41 ± 5.03	**< 0.001**	26.21 ± 3.51	28.74 ± 4.75	**< 0.001**	− 0.49 ± 0.91	− 0.67 ± 1.14	0.859
Smoking	375(31.2%)	15(3.3%)	**< 0.001**	167(18.6%)	3(0.9%)	**< 0.001**	208(38.4%)	12(66.6%)	**0.001**^**+**^
Physical Activity	5.64 ± 2.64	4.91 ± 2.22	**< 0.001**	6.77 ± 2.84	6.13 ± 2.63	**0.006**	1.13 ± 2.51	0.33 ± 2.41	**0.001**^**+**^
*Psychological*									
Quality of life − Emotional	4.91 ± 0.93	4.39 ± 0.97	**< 0.001**	5.07 ± 0.82	4.72 ± 0.89	**< 0.001**	0.16 ± 0.80	0.33 ± 0.81	**0.028**^**+**^
Quality of life − Social	4.98 ± 1.08	4.47 ± 1.10	**< 0.001**	5.43 ± 0.97	5.13 ± 1.07	**< 0.001**	0.45 ± 0.94	0.66 ± 0.99	**0.018**^**+**^
Quality of life − physical	4.80 ± 1.08	4.32 ± 1.9	**< 0.001**	5.34 ± 0.95	4.91 ± 1.04	**< 0.001**	0.54 ± 0.91	0.59 ± 0.91	**0.030**^**+**^
Anxiety	37.50 ± 9.44	43.58 ± 12	**< 0.001**	35.82 ± 8.87	42.21 ± 12.55	**< 0.001**	− 1.68 ± 7.68	− 1.37 ± 8.97	0.819
Depression	8.99 ± 7.85	10.84 ± 8.92	**< 0.001**	7.41 ± 7.68	3.17 ± 9.96	**< 0.001**	− 1.58 ± 5.83	− 7.67 ± 7.17	0.311
*Clinical*									
SBP (mmHg),rest	115.97 ± 17.79	118.35 ± 18.09	**0.047**	114.40 ± 16.91	115.47 ± 15.17	0.438	− 1.57 ± 18.88	− 2.88 ± 17.33	0.726
SBP (mmHg),max	130.50 ± 22.35	131.12 ± 24.73	0.674	71.84 ± 10.49	72.49 ± 9.80	0.330	− 58.66 ± 20.16	− 58.6 ± 81.73	0.299
DBP(mmHg),rest	71.84 ± 10.49	72.49 ± 9.80	0.330	71.34 ± 9.91	71.94 ± 9.36	0.465	− 0.50 ± 11.16	− 0.55 ± 11.08	0.625
DBP(mmHg),max	77.92 ± 10.73	78.98 ± 11.64	0.137	77.25 ± 11.06	79.03 ± 14.65	**0.051**	− 0.67 ± 12.24	− 0.05 ± 16.63	0.632
METs	9.41 ± 2.81	6.56 ± 2.29	**< 0.001**	12.30 ± 2.91	8.67 ± 2.45	**< 0.001**	2.89 ± 2.62	2.11 ± 1.82	**0.001**^**+**^
EF	49.86 ± 11.48	53.80 ± 11.06	**< 0.001**	52.85 ± 10.37	55.65 ± 10.25	**< 0.001**	2.99 ± 11.97	1.85 ± 7.44	0.617
*Lab data*									
FBS	106.63 ± 33.04	119.07 ± 46.44	**< 0.001**	104.12 ± 28.35	113.78 ± 40.99	**< 0.001**	− 2.51 ± 23.82	− 5.29 ± 41.19	0.321
LDL	109.83 ± 43.91	123.28 ± 47	**< 0.001**	103.37 ± 38.46	115.63 ± 42.71	**< 0.001**	− 6.46 ± 33.89	− 7.65 ± 40.35	0.501
HDL	39.31 ± 14.50	42.71 ± 9.90	**< 0.001**	39.54 ± 10.31	44.04 ± 9.68	**< 0.001**	0.23 ± 9.90	1.33 ± 10.10	0.150
TG	179.24 ± 98.59	201.25 ± 104.40	**< 0.001**	167.78 ± 89.72	190.54 ± 101.76	**< 0.001**	− 11.46 ± 85.18	− 10.71 ± 91.02	0.959
Cholesterol	186.48 ± 54.28	209.26 ± 57.44	**< 0.001**	176.65 ± 47.56	200.01 ± 52.91	**< 0.001**	− 9.83 ± 41.86	− 9.25 ± 49.93	0.954

Bold indicates significant result

Body Mass Index. Based on IPAQ score (International Physical Activity Questionnaire). SBP; systolic blood pressure, DBP; diastolic blood pressure, FBS; fasting blood sugar, LDL; low density lipoprotein, HDL; high density lipoprotein, TG; triglyceride

+ : Comparing the differences between pre- and post- CR in both male and female

#### Lifestyle

Females have a higher BMI and their reduction were higher than male, as well but this reduction was not significant (*P*-value: 0.859). Although the number of smokers was greater in males before CR sessions, the cessation of smoking was greater in females (66.6% compare with 38.4%). In terms of physical activity CR sessions cause more activity in male compared to female (1.13 ± 2.51 compare with 0.33 ± 2.41).

#### Psychological

Although all subtypes of QOL (emotional, social and physical) were greater in male at the beginning of CR, after finishing sessions female experienced much increased in these terms (0.33 ± 0.81, 0.66 ± 0.99, 0.59 ± 0.91 compare with 0.16 ± 0.80, 0.45 ± 0.94, 0.54 ± 0.91).

#### Clinical

The changes in SBP and DBP both rest and max were not significant. But in terms of METs: The initial METs was higher in males, also after finishing sessions it increased much better in males (2.89 ± 2.62 compare with 2.11 ± 1.82).

Lab data: All parameters’ changes were not significant.

In the crude and adjusted model that data are shown in Table 3, which did not account for smoking and quality of life variables, all subscales showed significant changes before and after in both women and men. Notably, the change in smoking status was 17.6% higher in women compared to men, despite a smaller sample size of female smokers. This finding suggests that women who smoke benefitted from the rehabilitation intervention, leading to an increase in smoking cessation.

**Table 3:** Sex effect (female = 1) on change of risk factors of disease using crude model and adjusted model

Variables	Crude				Adjusted			
	B	SE	CI 95%	*P*-value	B	SE	CI 95%	*P*-value
*Lifestyle*								
Δ BMI	0.035	0.065	(− 0.092, 0.162)	0.589	0.055	0.087	(− 0.17, 0.226)	0.531
Δ Smoking (better or without change)	− 1.735 (OR = 0.176)	0.396	(0.081, 0.384)	< 0.001	− 0.894 (OR = 0.409)	0.798	(0.086, 1.96)	0.263
Δ Physical activity	0.137	0.222	(− 0.299, 0.573)	0.537	0.153	0.262	(− .0360, 0.667)	0.557
*Psychological*								
Quality of life− Δ Emotional	0.146	0.067	(0.016, 0.277)	0.028	0.164	0.08	(0.007, 0.32)	0.040
Quality of life− Δ Social	0.188	0.079	(0.032, 0.344)	0.018	0.178	0.094	(0.006, 0.362)	0.058
Quality of life − Δ Physical	0.164	0.076	(0.016, 0.313)	0.030	0.169	0.090	(− 0.09, 0.346)	0.062
Δ Anxiety	0.135	0.588	(− 1.02, 1.29)	0.819	0.725	0.802	(− 0.85, 2.29)	0.266
Δ Depression	− 0.457	0.452	(− 1.344, 0.429)	0.311	− 0.065	0.63	(− 1.30, 1.17)	0.918
*Clinical*								
ΔSBP (mmHg), rest	− 0.589	1.61	(− 3.75, 2.57)	0.714	0.852	1.89	(− 2.86, 4.56)	0.652
ΔSBP (mmHg), max	3.67	3.56	(− 3.33, 10.66)	0.304	10.51	7.18	(− 3.60, 24.61)	0.144
ΔDBP (mmHg), rest	0.495	0.972	(− 1.41, 2.40)	0.611	0.595	1.15	(− 1.66, 2.85)	0.604
ΔDBP (mmHg), max	0.517	1.074	(− 1.59, 2.63)	0.630	2.46	1.78	(− 1.05, 5.96)	0.169
ΔMETs	− 0.72	0.162	(− 1.036, − 0.40)	< 0.001	− 0.926	0.246	(− 1.41, − 0.442)	< 0.001
ΔEF	− 0.78	2.43	(− 6.55, 2.99)	0.465	− 0.234	0.698	(− 1.61, 1.14)	0.738
*Lab data*								
ΔFBS	− 2.41	1.92	(− 6.179, 1.37)	0.211	2.04	2.61	(− 7.16, 3.08)	0.435
ΔLDL	− 1.78	2.43	(− 6.55, 2.99)	0.465	− 0.67	3.05	(− 6.66, 5.31)	0.826
ΔHDL	0.946	0.657	(− 0.34, 0.23)	0.150	0.76	0.899	(− 1.00, 2.53)	0.395
ΔTG	0.287	5.62	(− 10.73, 11, 30)	0.959	2.78	7.35	(11.66, 17.21)	0.71
Δ Cholesterol	0.179	2.86	(− 5.42, 5.78)	0.950	− 1.76	3.73	(− 9.08, 5.56)	0.638

Regarding quality of life, the scores improved more significantly in women than in men. The emotional score of women was 0.146 times higher than that of men, the social change score was 0.188 times higher, and the physical change score was 0.164 times higher.

However, after adjusting the model to consider smoking and quality of life variables, only emotional and Mets variables remained statistically significant (Table 3).

Adjustment was done by all variables in Table 1.

## Discussion

The main results of this study are: (1) Despite males were slightly more willing to continue the CR program than females, there were not a significant different. (2) Both men and women improved significantly after CR in terms of quality of life (MACNEW score) including emotional, physical, and social activities, BMI, LDL-C, and total cholesterol levels, (3) FBS and TG levels were improved more significantly in men than women. Our results have shown that the CR effects on risk factor modification and exercise ability are good for both all participants. Secondary findings of this study are differences in the patient's baseline characteristics, on the one hand, females had worse conditions in terms of socio-economic (women had worse marital status, and the number of women who were educated more than 5 years are lesser than men, also the majority of women were household) and comorbidities (females had more comorbidities such as DM, HTN, Hyperlipidemia than man), on the other hand, the number of females who are treated as PCI was lesser than males.

There is no doubt both home-based and technology-based CR programs can result in improving health-related quality of life and cardiac outcomes [7, 14] our study demonstrates that in terms of quality of life, females can benefit from CR programs as much as males can, which is consistent with previous studies [15–17]. Based on the socio-economic conditions of our participants in the CR program, it can be expected that a reduced number of females would be eager to participate in the CR program., this issue has been shown and discussed in many studies in different ways. For instance, Firoozabadi et al. compared the participation of women and men in depressed areas and higher-resource areas and concluded that not only did this group participate in CR programs less than in privileged areas, but also had different inhibiting factors. They divided the obstacles into two groups: Logistics which are related to financial issues (including distance, cost, insurance, and transportation problems), and functional status, which is related to individuals (including age, energic level, functional capacity) [1]. Moreover, Mamataz et al. summarized some barriers for females, not participating in CR programs as much as males, such as psychosocial reasons (depression and anxiety), transportation issues, and lower referral rates [18]. Interestingly Ritchey et al. showed that gender disparity in terms of enrolling in CR programs increases by age, race, and ethnicity and this increase is more highlighted in Asians [19]. Furthermore, Marzolini et al. counted family obligations, transportation, and musculoskeletal and multiple medical problems as the main reasons for female CR programs withdrawal [20]. It has been proven that there are many gender disparities among the Iranian population such as physical activity [21], education field [22], obesity [23] health outcomes and health care access [24]. Also, the results of previous studies in terms of CR programs have shown the extent of CR in different provinces of Iran, based on which this program is implemented more in developed provinces [25].

Our study showed that females suffer more from depression and anxiety than men and have a worse quality of life (BMI and Physical activity) in both pre and post-CR programs, Factors that have already been proven to have negative outcomes in CVDs. However, in some ways, females had better performance during CR than males such as quitting smoking. And these results are consistent with other previous studies such as Panzeri et al. evaluated the effect of CR programs in both genders and showed elder women have more disability and depression, so they have a worse quality of life than younger men, hence they mentioned, these qualities influence outcomes of CR programs like higher rate of mortality in women [26]. Some studies have even gone further and have shown that to obtain superior psychological results in certain patients such as those with depression and anxiety, more special psychological care should be chosen than the routine CR program [27].

Among the various clinical results, we chose METs and changes in BP and EF because these items not only indicate the cardiopulmonary condition but also play a role as risk factors.

Our study demonstrated that females were more prone to high BP and during the program, the amount of change in SBP Max was not significant in them, except for this, the CR program had a beneficial effect on the rest of the variables in both sexes. These results were consistent with the previous study. For instance, Quindry jc et al. demonstrated that The CR program, especially the second phase, is very effective and useful for controlling blood pressure, although this effect is different based on gender and underlying disease [28].

Generally, all participants had benefited from CR in terms of controlling cardiac comorbidities; however, FBS and TG have decreased more in males; however, other lipid profiles have decreased more in females than males, checking the participants’ baseline characteristics (Table 1), revealed that DM, HTN, and Hyperlipidemia have more prevalence among the female gender. Previous studies showed that CR program influences controlling cardiac risk factors so that it could have influenced cardiac mortality [29, 30].

Although in our study the CR program caused a decrease in most of the variables, after adjusting, it was found that the decreasing rate in METs and QOL emotions is only significant in both genders. This result is consistent with some previous studies such as meta-analyses like the study done by Bjarnason-Wehrens et al. in heart failure patients [31] or the clinical trial study which was done by Smith KM et all on CABG patients [32] Or interventional study on ischemic heart disease patients [33]. Therefore, by starting the CR sessions immediately before discharge from the hospital and informing the doctors and patients about its results, especially its benefits for female patients considering the pathophysiology of their disease, the participation rate can be increased.

In our study, we only compared participation in women vs men in the CR program, but we did not study the reasons. In the next step, we will study the reasons for women's non-participation, which can be systematic and social, such as lack of insurance or individual reasons, lack of access to a proper transportation system, or the presence of comorbidities. Moreover, because of longitudinal study some data can be missed.

## Conclusion

Based on this study, it can be concluded that the CR program can improve cardiovascular outcomes in three psychological, comorbid, and functional capacity fields, but the greatest impact was on the quality of life and patient METs. Also, in this study, the number of female participants in the CR program was less than the number of males, which was expected according to the basic demographic information and other gender disparities according to previous studies. More studies are needed to evaluate specific factors including these disparities based on low- middle-income countries such as Iran.

## Data Availability

The data that support the findings of this study are available from the corresponding author upon reasonable request.

## Abbreviations

BMI: Body Mass Index
BP: Blood Pressure
CABG: Coronary Artery Bypass Surgery
CR: Cardiac Rehabilitation
CVD: Cardiovascular Disease
DALYs: Disability-Adjusted Life Years
DBP: Diastolic Blood Pressure
DM: Diabetes Mellitus
EF: Ejection Fraction
FBS: Fasting Blood Sugar
HDL: High-Density Lipoprotein
HTN: Hypertension
IHD: Ischemic Heart Disease
IPAQ: International Physical Activity Questionnaire
LDL-C: Low-Density Lipoprotein Cholesterol
METs: Metabolic equivalents
MI: Myocardial Infarction
PCI: Percutaneous Coronary Interventions
QOL: Quality of life
SBP: Systolic Blood Pressure
TG: Triglyceride
